# Perinatal mortality after Chernobyl in former Soviet countries

**DOI:** 10.1371/journal.pone.0326807

**Published:** 2025-07-02

**Authors:** Alfred Körblein

**Affiliations:** Independent Researcher, Nuremberg, Germany; University of South Carolina, UNITED STATES OF AMERICA

## Abstract

A recent study examined trends in perinatal mortality (PM) in Ukraine for possible increases following the Chernobyl accident. PM rates from Belarus and other countries of the former Soviet Union (FSU) are analyzed here using essentially the same methods.

**Data and methods:**

Perinatal mortality data for the FSU countries are available in the Health for All database (WHO). In this study, data from Ukraine, Belarus, the Russian Federation, Moldova, and Estonia are analyzed. The regression model uses a long-term exponential trend with flexible time dependence and two superimposed bell-shaped terms (Model 1). In a second approach, the bell-shaped excess terms are replaced by the inverse of gross domestic product per capita (the GDP term), which serves as a proxy for the possible impact of the socio-economic crisis after the collapse of the Soviet Union. The possible strontium exposure of pregnant women (the strontium term) is added as a second covariate (Model 2).

**Results:**

Model 1 fitted the data of all five countries well. The observed increases in PM rates in the 1990s were greater in Belarus than in Ukraine and Russia. Model 2 regressions also fit the data well, except for Ukraine. In Belarus, Russia, and Moldova, the GDP term alone explained the deviation of PM rates from the predicted trend; adding the strontium term did not significantly improve the fit. Only in Ukraine and Estonia was the effect of the strontium term statistically significant. In 1987, increases in PM were found in all countries except Estonia, where PM peaked in 1988.

**Conclusion:**

The deviation of PM rates in the 1990s from the long-term trend is related to GDP per capita. An effect of the strontium term is detected only in Ukraine and Estonia, where sharp increases in PM were observed in the early 1990s, well before the trough of GDP in the second half of the 1990s.

## Background

Recently, an increase in perinatal mortality in Ukraine during the 1990s was reported, superimposed on a long-term decreasing trend [1]. The deviation of perinatal mortality from the predicted trend was approximately three times higher in Ukraine’s three most contaminated oblasts (Zhytomyr, Kyiv, and the city of Kyiv) than in the rest of the country. To corroborate these findings, the present study analyzes perinatal mortality data from Belarus, the country most affected by the Chernobyl accident. Due to the population’s higher average radiation exposure, the increase in the 1990s should be greater in Belarus than in Ukraine.

In 2000, at a conference in Minsk, I obtained official data on perinatal mortality in Belarus from 1985 to 1998 from the Belarusian Ministry of Health. Oblast-level data was also provided. Data on perinatal mortality for the years 1993–2019 are available in the Health for All database of the World Health Organization (WHO) [2]. Combining these two datasets enables analysis of Belarusian data from 1985 onwards. In the present study, the results for Belarus are compared with those for Ukraine and Russia.

## Materials and methods

The Health for All database contains perinatal mortality data for birth weights of 1000 g and above, including the number of live births (LB), early neonatal deaths (NEO), and perinatal deaths per 1000 births (PM). Stillbirths (SB) are not included but can be calculated using the perinatal mortality definition: PM = (SB + NEO)/ (LB + SB). Mind that the definitions of live births, stillbirths, and early neonatal deaths differed in the Soviet Union from the WHO definitions. According to the recommendations of the World Health Organization, “a live birth occurs when the infant is expelled or extracted from the mother, regardless of the length of the pregnancy, after which the child breathes or shows any other sign of life, such as heartbeat, pulsation of the umbilical cord, or voluntary muscular movement.” The Soviet definition of a live birth differed from that recommended by WHO. The only sign of life considered was whether the newborn breathed; other signs of life were ignored. Furthermore, according to the Soviet definition, “children who are born before 28 weeks of gestation have been completed, or who weigh less than 1000 grams, or who are less than 35 centimeters in length, are not supposed to be counted as either live births or infant deaths if they die within the first seven days (168 hours) after delivery, whether or not they ever took a breath. Instead, they are counted as miscarriage” [3]. According to [3], by applying the WHO definitions instead of the Soviet definition, the infant mortality rates would increase by 22–25 percent.

For this study, the differences in the definition of perinatal deaths are irrelevant as long as they do not change during the study period. Level changes account for any changes in the long-term trend of perinatal mortality.

### Regression Model 1

To analyze the longer period of data available in the HFA database, the linear exponential trend used in [1] is replaced by an exponential trend with a third-degree polynomial in time. The observed deviations from the long-term trend are fitted with two superimposed bell-shaped excess terms of equal half-width. The regression model (Model 1) is as follows:


$$PM(t)\;\sim \;{exp(\beta }_{1}\;+\beta_{2}\cdot t+\;\beta_{3}\cdot t^{2}+\beta_{4}\cdot t^{3}{+\;\beta }_{5}/t/exp((log(t)-\;\beta_{6}\hat{)}2/2/\beta_{7}^{2})+\;\beta_{8}/t/exp((log(t)-\;\beta_{9}\hat{)}2/2/\beta_{7}^{2}))$$

In Equation 1, PM(t) denotes perinatal mortality, where t is the calendar year minus 1980. The parameter β₁ is the intercept, while β₂ through β₄ are trend parameters. Lognormal density distributions are used for the bell-shaped excess terms (parameters β₅ to β₉). Parameters β₅ and β₈ estimate the effect sizes, while β₆ and β₉ estimate the logarithms of the medians. The parameter β₇ estimates the common standard deviation. Iterative reweighted non-linear regression is applied using the nls() function of the R statistical package [4]. The weights are 1/var, where var is the binomial variance, var= fit·(1 − fit)/(LB + SB), with fit denoting the fitted values. F-tests and two-tailed t-tests are used to determine statistical significance, with a p-value of less than 0.05 being considered significant.

The number of excess cases associated with the first and second excess terms is estimated as sum((LB+SB)·(fit−fit2)) and sum((LB+SB)·(fit2−fitr)). Here, fit refers to the fitted model; fitr refers to the reduced model, i.e., the long-term trend without the bell-shaped excess terms; and fit1, fit2 refer to the first and second bell-shaped terms, respectively. The time at which the bell-shaped terms peak is calculated using exp(b6+b72)+1980 and exp(b9+b72)+1980, for the first and second terms, respectively. The size of the bell-shaped excess terms is determined by max((fit−fit2)/fit2) and max((fit2−fitr)/fitr), respectively.

### Regression Model 2

In Model 2, the bell-shaped excess terms are replaced by the inverse of gross domestic product (GDP) per capita and the calculated strontium concentration in pregnant women, as described in [1]. Since the regression model is linear, logistic regression is applied for the analysis with Model 2. The R function glm(family=quasibinomial) is used to adjust the results for overdispersion.

**Fig 1:**
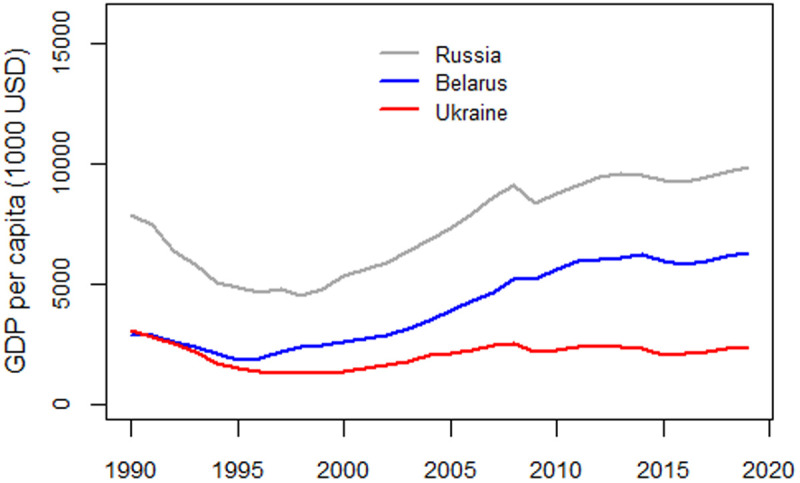
Development of GDP per capita in Russia, Belarus, and Ukraine, 1990-2019, expressed in units of 1000 2015 US dollars (data from worldbank.org).

GDP per capita serves as a proxy for the potential effects of the socioeconomic crisis that followed the collapse of the Soviet Union. World GDP data is available on the World Bank’s website [5]. Fig 1 illustrates GDP per capita trends in the Russian Federation, Ukraine, and Belarus, expressed in constant 2015 U.S. dollars. In all three countries, GDP declined after 1991, reaching its lowest value in the second half of the 1990s.

In a 2004 paper, Körblein hypothesized that strontium exposure may explain the trend in perinatal mortality in West Germany after atmospheric nuclear weapons testing in the 1950s and early 1960s [6]. In 2024, Körblein applied his strontium model to analyze the ratio of perinatal mortality rates in the most contaminated regions of Ukraine to the rates in the rest of the country [1].

The strontium hypothesis is explained in detail in references [1] and [6], but will be outlined briefly below. Radioactive strontium replaces calcium in bones. The greatest uptake of strontium occurs during the period of greatest bone growth. For girls, this occurs around age 14. The concentration of strontium in a group of pregnant women in the years following Chernobyl depends on the proportion of women who were 14 years old during the year of peak dietary strontium concentration (i.e., 1986). This proportion is determined by the maternal age distribution. Strontium excretion from the body must also be considered. A rate of 2.6% per year is used, as proposed in [7], for women between 20 and 40 years old. Strontium-90 irradiates the red bone marrow, weakening the immune system [8]. This may lead to an increased perinatal mortality rate.

**Fig 2:**
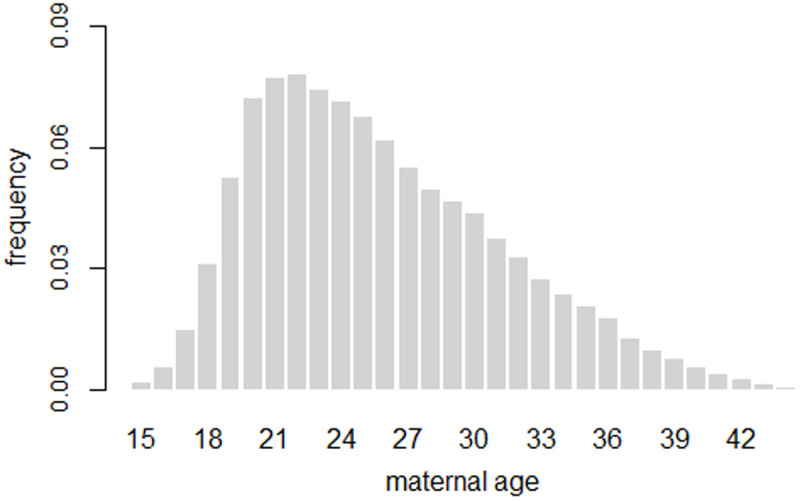
Maternal age distribution from St. Petersburg averaged during 1990-1999.

Maternal age distributions on an annual basis were not available for the countries investigated in this paper. However, at a conference in St. Petersburg in 2001, Körblein obtained annual maternal age distributions from St. Petersburg for the years 1990–1999, see Fig 2. These data are used in this study.

Measurements of strontium content in pregnant women after Chernobyl were not available. However, such measurements were made on inhabitants of settlements along the Techa River in the Southern Urals region of Russia. From 1949 to 1956, liquid waste containing fission products from the Mayak plutonium production complex was released into the Techa River and its floodplains. The river was the main water supply for drinking and other uses. From 1949 to 1956, the average ingestion levels of Sr-90 for inhabitants of villages in the mid-Techa region (70–140 km downstream from the release site) amounted to 3,150 kBq, 95% of which was ingested from 1950 to 1952.

**Fig 3:**
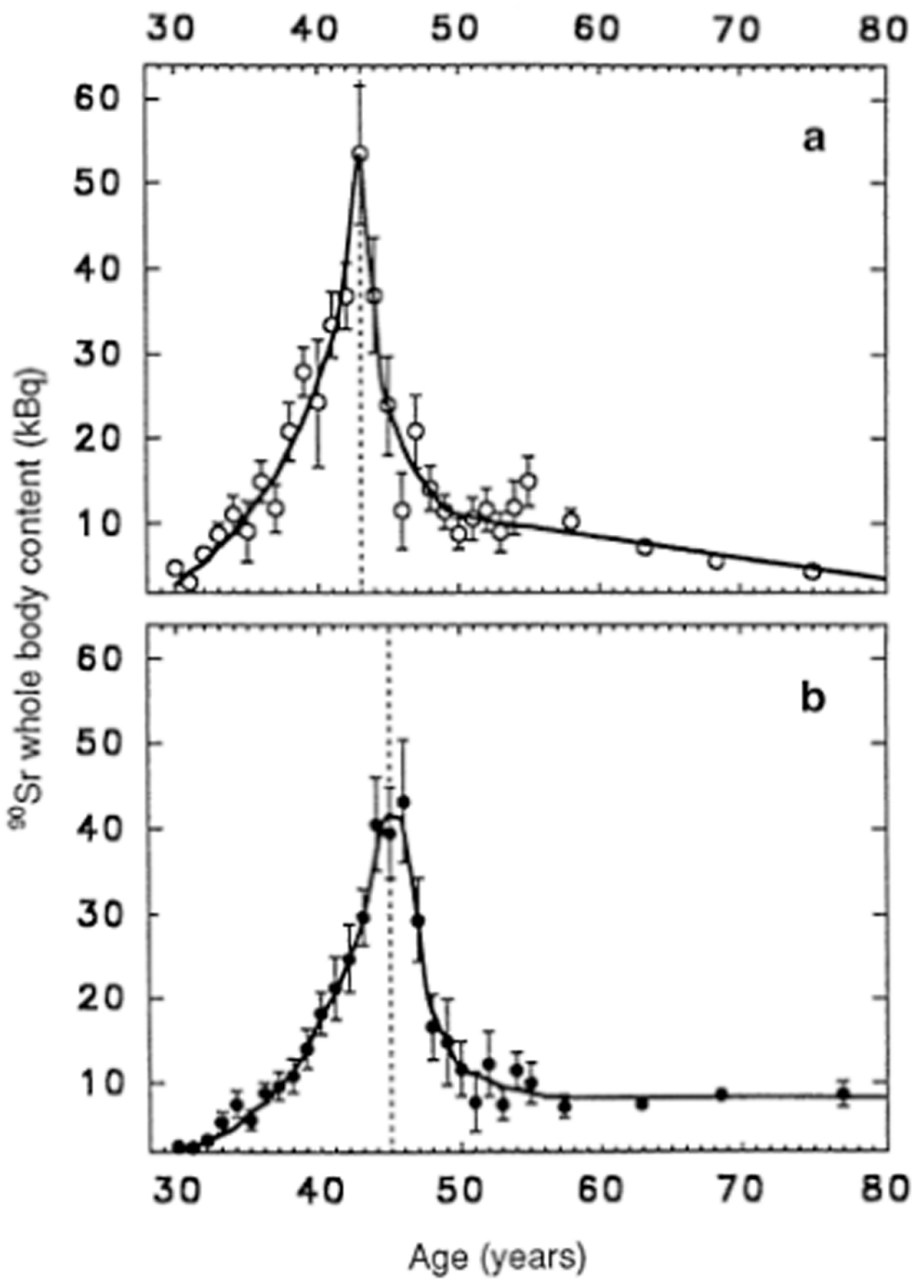
Average values of 90Sr whole-body content for different age cohorts of Muslyumovo residents in 1980 (a: women, b: men). Vertical bars represent standard deviations. From [7] Fig 2.

Fig 3, top panel, shows the Sr-90 content (kBq) in female (top panel) and male (bottom panel) residents of the village of Muslyumovo as a function of age, measured in 1980. In females, the strontium content peaked at 43 years of age. Assuming that 1950 was the year of maximum release, women 43 years old in 1980 were 13 years old in 1950. Thus, these results support the strontium hypothesis.

## Results

### Ukraine

**Fig 4:**
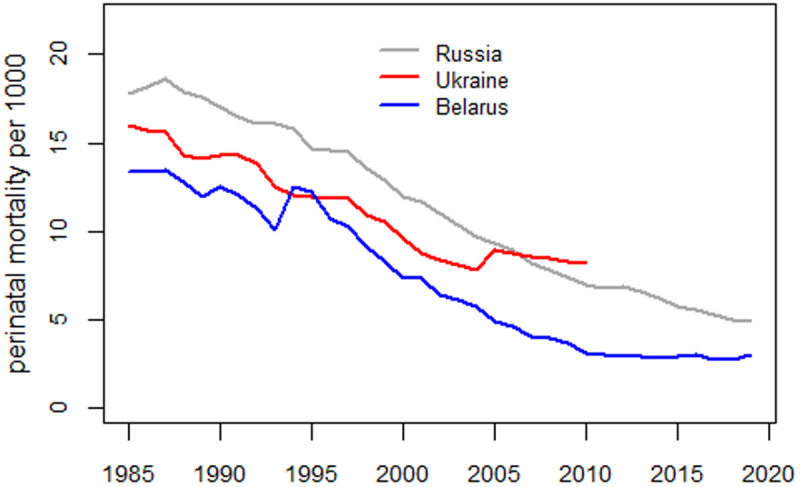
Perinatal mortality trends in Ukraine, Belarus, and the Russian Federation.

Fig 4 shows perinatal mortality trends in Ukraine, Belarus, and the Russian Federation. HFA data for Ukraine are available for the period 1981–2010, while the data from the Ministry of Health of Ukraine used in [1] ended in 2006.

**Fig 5:**
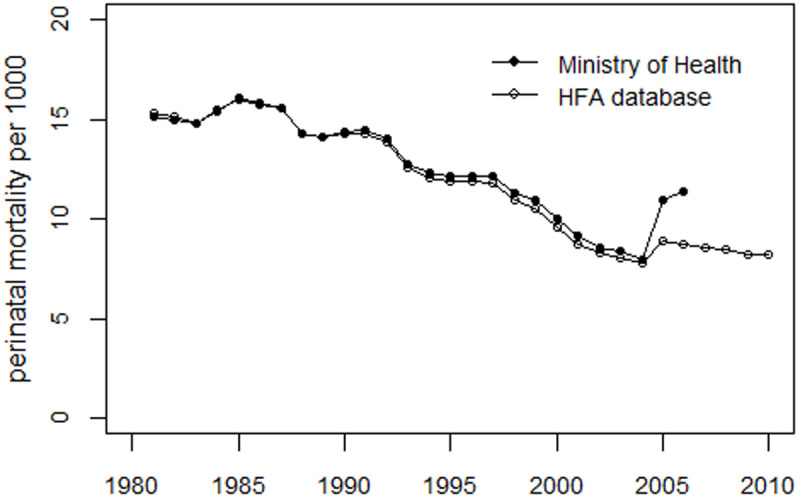
Comparison of perinatal mortality rate in Ukraine using data from the HFA database and the Ministry of Health of Ukraine.

To verify the compatibility of the HFA database data with the Ministry of Health of Ukraine data, the Ukrainian HFA data are first analyzed using the regression model in [1]. Table 1 compares the HFA data with the Ministry of Health’s data, and Fig 5 illustrates the trends in perinatal mortality rates.

**Table 1:** Perinatal mortality data from the HFA database and the Ministry of Public Health.

Year	Ministry of Public Health of Ukraine				HFA database			
	LB	SB	NEO	Rate	LB	SB	NEO	Rate
1985	762775	7841	4548	16.08	759144	7699	4563	15.99
1986	792574	7873	4784	15.81	781923	7737	4700	15.75
1987	760851	7504	4448	15.56	751006	7379	4444	15.59
1988	744056	6710	4007	14.27	729792	6566	3957	14.29
1989	690981	6143	3723	14.15	680007	5961	3691	14.07
1990	657202	5724	3815	14.39	647921	5597	3722	14.26
1991	630813	5338	3861	14.46	624221	5237	3771	14.31
1992	596785	4818	3613	14.01	596025	4736	3579	13.84
1993	557467	3990	3181	12.77	552839	3867	3125	12.56
1994	521545	3707	2749	12.29	520865	3589	2736	12.06
1995	492861	3409	2637	12.18	494086	3326	2608	11.93
1996	467211	3218	2511	12.18	473333	3146	2510	11.87
1997	442581	2966	2456	12.17	443366	2876	2403	11.83
1998	419238	2597	2149	11.25	414875	2502	2064	10.94
1999	389208	2353	1927	10.93	386679	2244	1855	10.54
2000	385126	2076	1813	10.04	386744	1976	1740	9.56
2001	376478	1830	1623	9.13	374576	1750	1547	8.76
2002	390688	1837	1530	8.58	383734	1739	1476	8.34
2003	408589	1969	1465	8.36	401810	1862	1396	8.07
2004	427259	1986	1425	7.95	413958	1876	1376	7.82

LB: Live births; SB: Stillbirths; NEO: Early neonatal deaths

To confirm the main results reported in [1] for Ukraine, a regression analysis of perinatal mortality was conducted using HFA data from 1985 to 2004. The regression model applied in [1] consists of a long-term exponential trend with two superimposed bell-shaped excess terms and a dummy variable (d87) for 1987. This was the year after the Chernobyl accident when a statistically significant increase in perinatal mortality was observed in Germany [9].

The model fitted the HFA data well. The deviance was 11.8 (df = 12) with the two bell-shaped excess terms and 290.1 (df = 17) without them. This improvement in fit was highly statistically significant (p = 6.2E-8, F-test). The regression results (parameter estimates, standard errors of the estimates, and t-values) are shown in Table 2, alongside the corresponding results from the regression using data from the Ministry of Health. All parameter estimates are within the error limits. Thus, the main results of [1] are confirmed with the HFA data.

**Table 2:** Regression results for Ukraine using Ministry of Health and HFA data.

Parameter	Variable	Ministry of Health			HFA database		
		Estimate	SE^1^	t-value	Estimate	SE	t-value
β_1_	intercept	−3.936	0.009	−454.17	−3.931	0.008	−500.63
β_2_	t	−0.038	0.001	−44.96	−0.039	0.001	−54.17
β_5_	magnitude	1.413	0.112	12.57	1.404	0.104	13.51
β_6_	^2^mu	2.445	0.011	218.31	2.441	0.010	244.69
β_7_	^3^sigma	0.121	0.008	14.68	0.115	0.007	15.49
β_8_	magnitude	2.816	0.228	12.33	2.547	0.200	12.72
β_9_	mu	2.864	0.011	251.51	2.852	0.011	270.46
β_11_	d87	0.037	0.011	3.30	0.043	0.010	4.14

^1^SE = standard error of estimate;

^2^mu = ln(median),

^3^sigma = standard deviation of lognormal distribution

**Fig 6:**
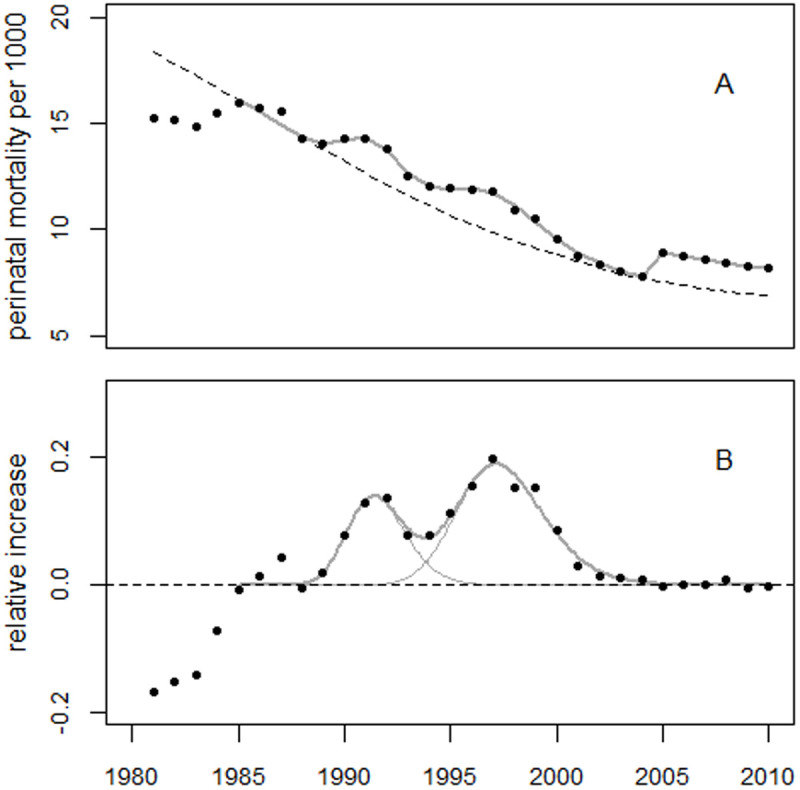
Panel A: Trend in perinatal mortality rates from Ukraine using data from the HFA database and regression line. The dashed line shows the predicted unperturbed trend. Panel B: Deviations between observed and predicted rates in relative units.

**Fig 7:**
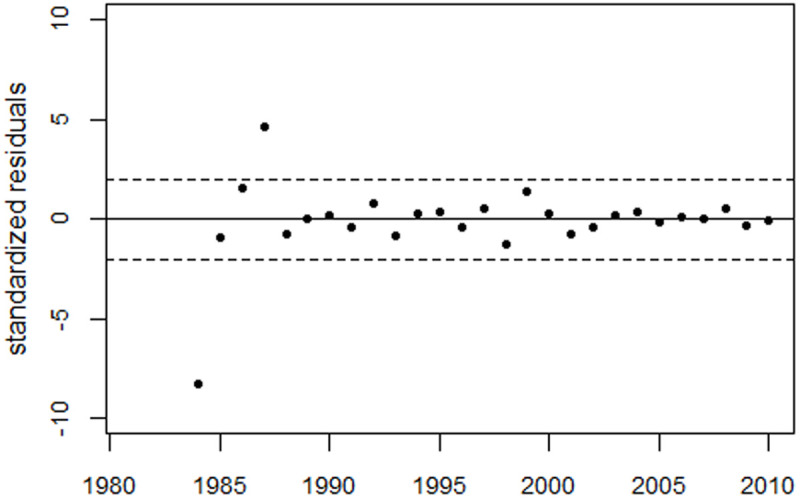
Deviations between observed and fitted perinatal mortality rates in units of standard deviation (standardized residuals).

For the extended period up to 2010, the regression model (1) was supplemented with dummy variables for the years 1981–1984, as well as a level shift in 2005. The model fitted the data well. The deviance was 10.7 (df = 15), whereas the regression without the bell-shaped terms (the reduced model) yielded a deviance of 176.2 (df = 20). The improvement in fit was highly statistically significant (p = 1.3E-8). Fig 6 shows the perinatal mortality rates and the regression line. Fig 7 shows the residuals in units of standard deviation (SD).

Table 3 displays the regression results, including the parameter estimates, standard errors of the estimates (SE), and t and p values.

**Table 3:** Regression results for Ukraine using HFA data (1981-2010).

Parameter	Variable	Estimate	SE^1^	t-value	p-value
β_1_	intercept	−3.969	0.039	−101.26	<2E-16
β_2_	t	−0.026	0.009	−2.77	0.0142
β_3_	t2	−1.3E-03	5.5E-04	−2.45	0.0269
β_4_	t3	3.6E-05	9.9E-06	3.68	0.0022
β_5_	height	1.496	0.155	9.68	7.7E-08
β_6_	^2^mu	2.448	0.009	282.66	<2E-16
β_7_	^3^sigma	0.121	0.008	15.67	1.1E-10
β_8_	height	2.997	0.241	12.43	2.7E-09
β_9_	mu	2.858	0.009	328.79	<2E-16
β_11_	cp05	0.170	0.016	10.77	1.9E-08
β_12_	d87	0.042	0.009	4.49	0.0004
β_12_	d81	−0.185	0.032	−5.85	3.2E-05
β_12_	d82	−0.164	0.024	−6.75	6.6E-06
β_12_	d83	−0.153	0.018	−8.48	4.2E-07
β_12_	d84	−0.075	0.013	−5.65	4.6E-05

^1^SE = standard error of estimate;

^2^mu = ln(median),

^3^sigma = standard deviation of lognormal distribution

The perinatal mortality rates from 1981 to 1984 are inconsistent with the data trends from 1985 to 2010. The likely reason for this discrepancy is underreporting before Gorbachev’s glasnost policy

The estimated level shift in 2005 was + 18.6% (95% CI: 12.3% to 22.7%), with a p-value of 1.9E8. The first and second bell-shaped terms peaked in 1991.4 and 1997.2, respectively, with increases of 13.3% and 18.9%. The estimated number of excess perinatal deaths associated with the first and second excess terms were 3,606 and 4,163, respectively. The 1987 peak was highly statistically significant, with an increase of 4.3% (90% CI: 2.6% to 6.0%), p = 0.0004. This corresponds to 487 (90% CI: 296–682) excess perinatal deaths.

#### Regression with Model 2.

Using logistic regression with only the inverse of GDP (GDP term) as a covariate reduced the deviation from 176.2 (df = 20) to 121.6 (df = 19). This improvement in fit is statistically significant (p = 0.009, F-test).

Regression with only the strontium term for age 14 at exposure in 1986 yielded a deviation of 138.1 (df = 19), a significant improvement in fit over the regression without the strontium term (p = 0.036).

A regression analysis using the terms GDP and strontium for age 14 at exposure yielded a deviance of 105.1 (df = 18) and a p-value of 0.110. For age 15 at exposure, however, the deviance was 87.3 with a p-value of 0.016. Age 15 at exposure corresponds to a one-year shift in the maternal age distribution. This seems plausible, as the maternal age distribution in St. Petersburg may not accurately represent that of Ukraine as a whole.

**Fig 8:**
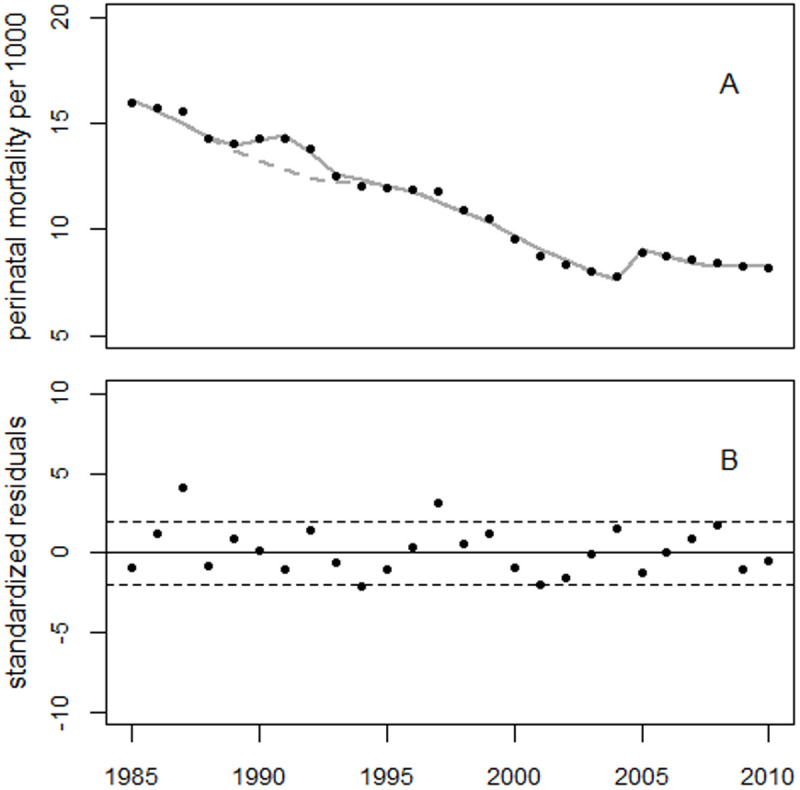
Top panel: Trend in perinatal mortality in Ukraine and the result of regression with both the inverse GDP and a bell-shaped excess term. Bottom panel: Residuals in units of standard deviation.

Using a regression with both the GDP term and a bell-shaped term to fit the observed peak around 1991 reduced the deviance from 121.8 (df = 19) to 40.6 (df = 16), with a p-value of 0.0004. Fig 8 shows the results of this regression and the residuals. However, with a deviance of only 10.7 (df = 15), regression with Model 1 yielded a much better fit (p = 1.0E-5). (Compare the residuals in Fig 8 with those in Fig 7).

The difference in trends between the full model (gray line in Panel A) and the reduced model (without the bell-shaped term) estimated 2,845 excess perinatal deaths associated with the bell-shaped term. For comparison, 3,606 excess cases were associated with the first bell-shaped term in Model 1.

### Belarus

#### Regression with Model 1.

Table 4 compares the number of stillbirths, early neonatal deaths, and perinatal mortality rates (PMR) published by the Ministry of Health (MOH) of Belarus with the corresponding HFA database data from 1993 to 1998. The MOH did not provide data on live births (LB), so it had to be calculated from stillbirths (SB), early neonatal deaths (NEO), and perinatal mortality (PM) using the perinatal mortality definition. Similarly, since stillbirths are not included in the HFA data, they were calculated using LB, NEO, and PM.

**Table 4:** Comparison of HFA data from Belarus with data from the Ministry of Health.

Year	Ministry of Health				HFA database			
	SB	NEO	PM	LB (calc)	SB (calc)	NEO	PM	LB
1993	595	594	10.08	117,361	595.2	594	10.08	117,384
1994	715	673	12.48	110,502	715.1	673	12.47	110,599
1995	645	599	12.22	101,155	644.9	599	12.22	101,144
1996	521	509	10.69	95,830	520.6	509	10.69	95,798
1997	526	401	10.29	89,561	528.0	401	10.29	89,754
1998	471	381	9.15	92,643	472.6	381	9.15	92,819

LB: Live births; SB: Stillbirths; NEO: Early neonatal deaths; peri: perinatal mortality rates

The reported perinatal mortality rates and SB and NEO counts are nearly identical in the two datasets. However, they differ slightly from the live birth figures in the HFA dataset. Therefore, the dataset used for the analysis is a combination of official Belarusian data from 1985 to 1992 and HFA data from 1993 to 2019.

The regression model (1) was supplemented once more with a dummy variable for 1987. Two additional dummy variables were employed to account for an outlier in 1993 and to determine the statistical significance of the observed peak between 1990 and 1991.

The regression model fitted the data well. Table 5 shows the improvement in model fit at each level of refinement. The dispersion factor, denoted OD (for overdispersion), is the deviance divided by the degrees of freedom (df2), which is a measure of the model’s goodness of fit. OD > 1 indicates overdispersion. The last two columns of Table 5 show the F-values and their corresponding p-values.

**Table 5:** Improvement in model fit with each level of model refinement.

Level	Variables	dev1	df2	OD	df1	F-value	p-value
0	Intercept, t	574.3	33	17.40			
1	t2, t3	159.1	31	5.13	2	40.44	2.3E-09
2	excess1	74.6	28	2.66	3	10.58	8.0E-05
3	excess2	48.2	26	1.85	2	7.13	0.0034
4	d93	25.1	25	1.00	1	23.00	6.3E-05
5	d87	23.9	24	1.00	1	1.15	0.29
6	d9091	17.2	23	0.75	1	9.08	0.006

Table 6 presents the parameter estimates, standard errors of the estimates (SE), t-values, and p-values.

**Table 6:** Regression results for perinatal mortality rates in Belarus.

Parameter	Variable	estimate	SE^1^	t-value	p-value
β_1_	intercept	−4.368	0.063	−69.44	<2.0E-16
β_2_	t	0.038	0.013	2.90	0.008
β_3_	t2	−5.7E-03	6.9E-04	−8.28	2.4E-08
β_4_	t3	9.7E-05	1.1E-05	8.75	8.8E-09
β_5_	magnitude	5.079	0.491	10.34	4.0E-10
β_6_	mu^2^	2.744	0.014	191.3	<2.0E-16
β_7_	sigma^3^	0.149	0.013	11.54	4.8E-11
β_8_	magnitude	5.202	0.901	5.77	7.0E-06
β_9_	mu	3.131	0.028	110.3	<2.0E-16
β_11_	d93	−0.166	0.032	−5.21	2.8E-05
β_12_	d87	0.037	0.021	1.78	0.088
β_13_	d9091	0.063	0.021	3.02	0.006

^1^SE = standard error of estimate;

^2^mu = ln(median),

^3^sigma = standard deviation of lognormal distribution

**Fig 9:**
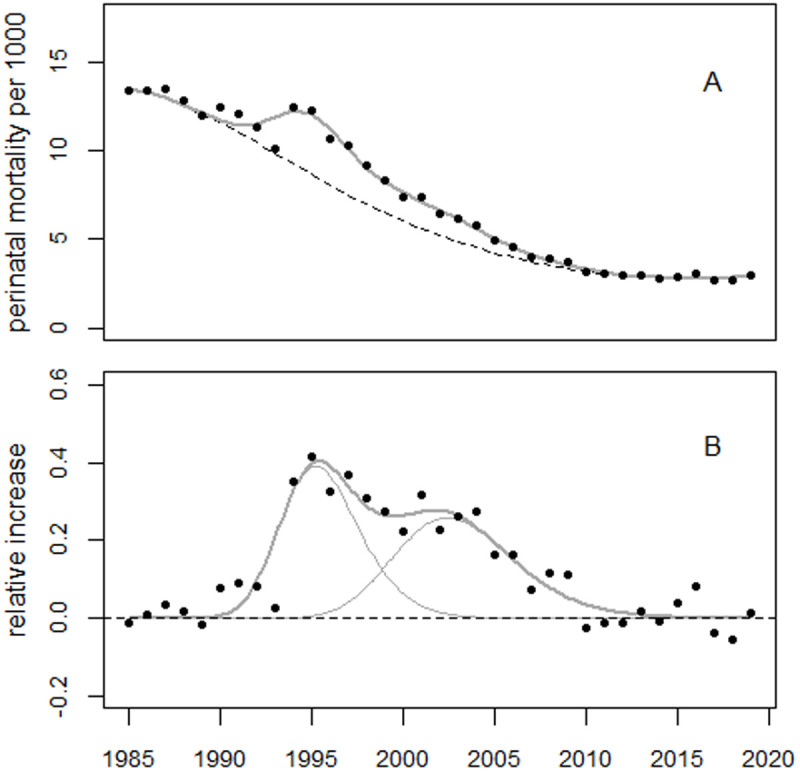
Trend in perinatal mortality rates in Belarus (Panel A) and deviations between the observed rates and the predicted trend in relative units (Panel B).

**Fig 10:**
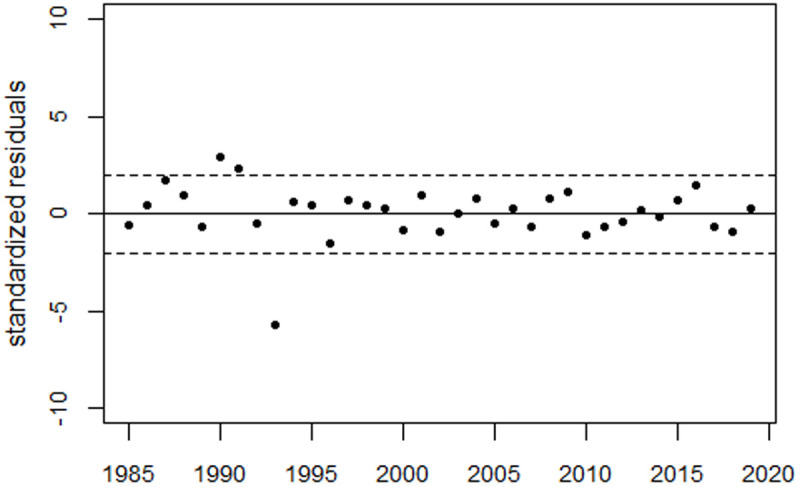
Trend of residuals in units of standard deviation (standardized residuals). The 1993 data point is an outlier.

Fig 9 illustrates the trend of perinatal mortality rates in Belarus, as well as the deviations between the observed rates and the predicted trend, expressed in relative units; Fig 10 shows the residuals. The 1993 data point is considered an outlier (p < 0.0001) and was excluded from the regression.

The peaks of the bell-shaped curves occurred in 1995 and 2002, respectively, with increases of 39% and 26%. The two excess terms were associated with 1,895 and 984 excess perinatal deaths, respectively. The increase in 1987 was estimated to be 3.7% (p = 0.088) which corresponded to 80 (90% CI: 2–158) excess perinatal deaths. The increase in 1990–91 was 6.5% (p = 0.006) which corresponded to (90% CI: 88–330) in 1990–1991.

#### Regression with Model 2.

**Fig 11:**
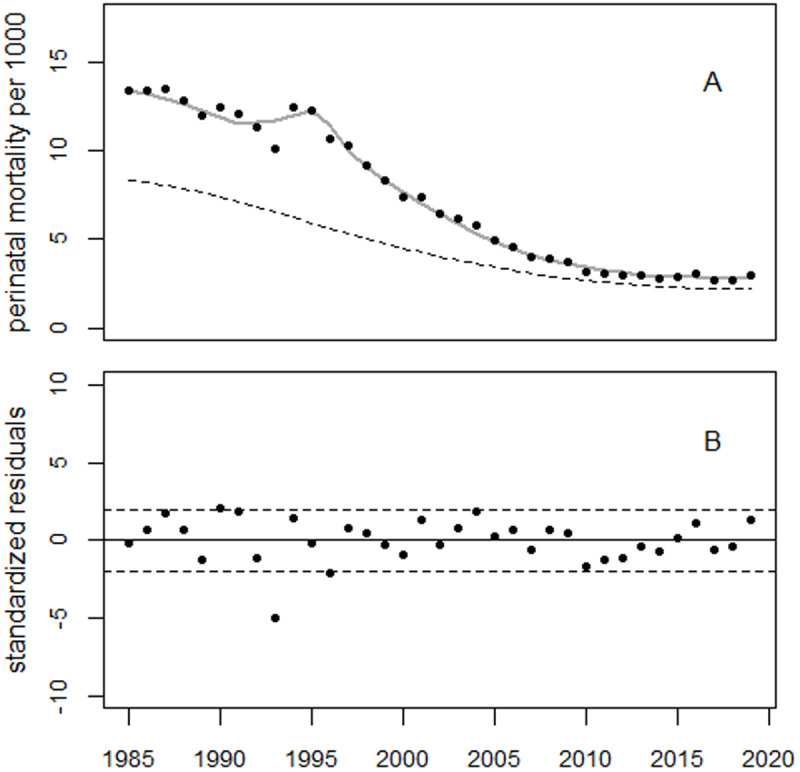
Upper panel: Trend of perinatal mortality in Belarus and the result of the regression with the GDP term. The dashed line shows the reduced model. Bottom panel: Residuals in units of standard deviation.

Using logistic regression with the GDP term reduced the deviance from 114.0 (df = 28) to 29.2 (df = 27), a highly significant improvement in fit (p = 1.8E-9). Adding the strontium term for age 15 at exposure in 1986 reduces the deviance from 29.2 to 26.8 (p = 0.14), but the estimate of the strontium term has a negative sign. Fig 11 shows the trend of perinatal mortality in Belarus and the results of the regression analysis including only the GDP term, and the residuals.

#### Gomel oblast.

In addition to data on Belarus as a whole, the Ministry of Health also supplied perinatal mortality data for six oblasts and the city of Minsk. Thus, the perinatal mortality trend in the most contaminated oblast, Gomel, could be compared to the trend in the rest of Belarus [10].

**Fig 12:**
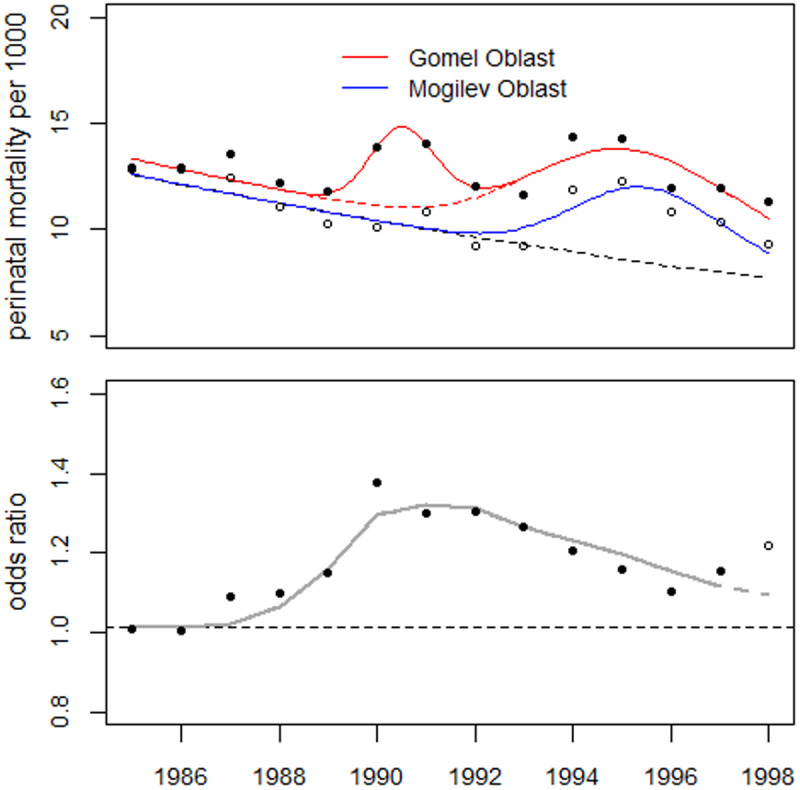
Top panel: Perinatal mortality trends in Gomel and Mogilev oblasts and regression lines. Lower panel: Ratio of perinatal mortality rates in the Gomel oblast to rates in the Mogilev oblast (odds ratios) as well as the result of regression using the strontium term to the power of 2.04. (bottom panel) illustrates the difference in perinatal mortality trends between the Gomel and Mogilev oblasts using the ratio of the perinatal mortality rates (odds ratios) in the two oblasts. The trend in the odds ratios was analyzed using the strontium term as a covariate. Regression analysis with exposure ages of 14, 15, and 16 in 1986 yielded deviances of 7.36, 3.68, and 1.57, respectively. An age of 16 at exposure is unrealistic; however, shifting the maternal age distribution by two years to younger ages corresponds to an age of 14 in 1986. The maternal age distribution in rural Gomel Oblast may peak at younger ages than in St. Petersburg.

Mogilev Oblast, which borders Gomel Oblast to the north, was the second most contaminated oblast by cesium-137 but much less so by strontium-90. Strontium is less volatile than cesium; therefore, strontium fallout was limited to areas closer to the Chernobyl reactor. A map of the surface ground contamination by strontium-90 is provided by the International Atomic Energy Agency [11]. Fig 12 (top panel) shows trends in perinatal mortality in the two oblasts, with results from combined regression analysis using a common slope and median for the bell-shaped excess that peaked in 1995–1996. In Gomel Oblast, a highly significant 24% increase was observed as early as 1990–1991 (p = 0.0002), whereas in Mogilev Oblast, no such increase was observed. A regression with a bell-shaped term to fit the increase from 1990 to 1991 significantly improves the fit of the model compared to a model without this term (p = 0.012). Additionally, 9.7% and 6.2% increases in perinatal mortality were found in the Gomel and Mogilev oblasts, respectively, in 1987.

According to the ICRP Publication 90, teratogenic effects are characterized by a sigmoid dose-response curve [12]. At low doses, this curve can be approximated by a power function (dose to a variable power n) [13]. A power of 1.8 ± 0.3 was found in [6] for the dependency of perinatal mortality in West Germany after the atmospheric nuclear weapon testing on the calculated strontium concentration in mothers.

Regression analysis using a power function for the strontium term yielded an exponent of n = 2.03 (95% confidence interval: 1.05–3.74). The trend line in Fig 12 lower panel shows the result of regression with the strontium term to the power of 2. The 1998 data point is significantly increased relative to the trend of the remaining data (p = 0.033). It was omitted from the regression analysis to avoid distorting the power estimate. The number of excess perinatal deaths in the years 1987−1997 in Gomel Oblast is estimated to be 416 (95% CI: 327–506), p = 7.0E-6

### Russia

#### Regression with Model 1.

**Fig 13:**
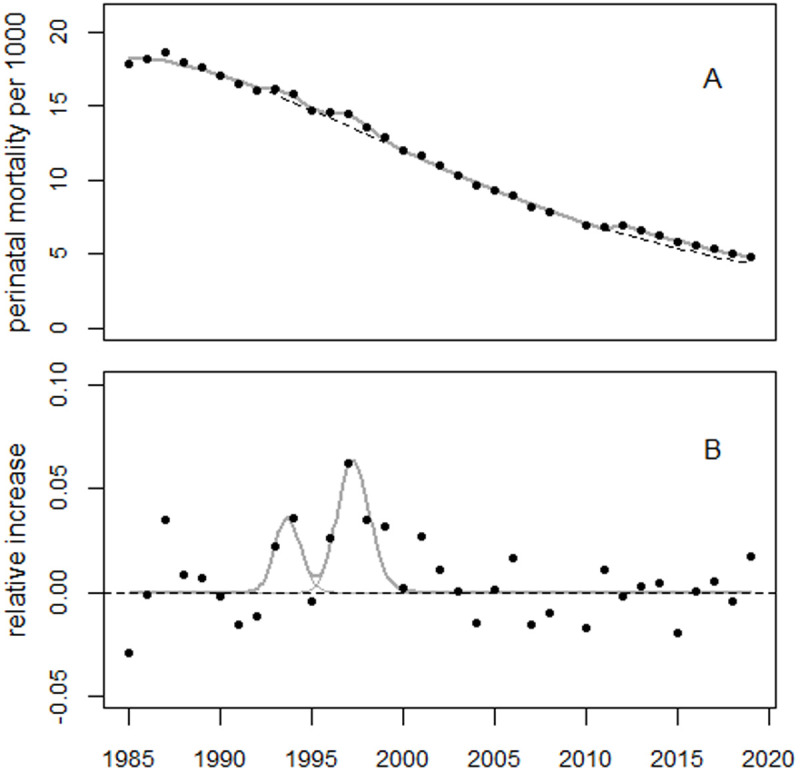
Trend in perinatal mortality in Russia and the result of regression using Model 1. The dashed line shows the predicted unperturbed trend. Panel B: Deviations from the trend in relative units.

**Fig 14:**
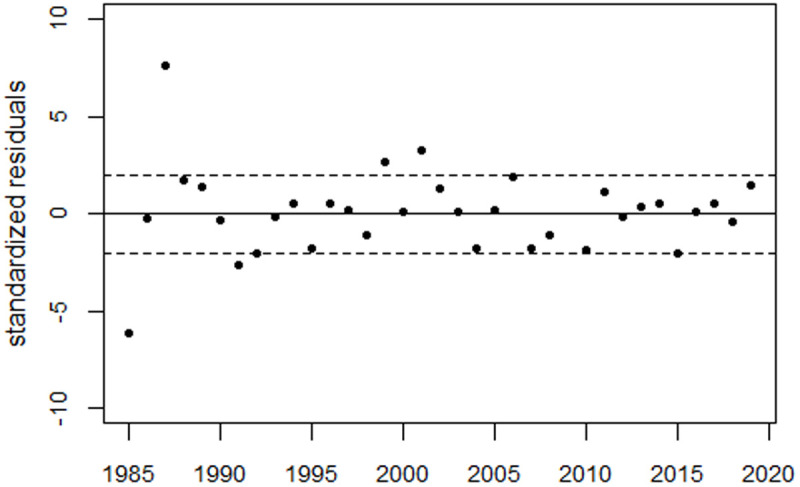
Deviations between observed and fitted perinatal mortality rates from Russia in units of standard deviation (standardized residuals).

To analyze the trend in perinatal mortality in the Russian Federation using model (1), dummy variables for 1985 (d85) and 1987 (d87) were added, as well as a level shift in 2012 (cp12). The model fitted the data well, the bell-shaped excess terms reduced the deviance from 143.6 (df = 27) to 64.5 (df = 22), p = 0.002 (F-test). The peaks of the excess terms are at 1993.7 and 1997.4, with increases of 3.2% and 6.1%, respectively. 1,367 and 2,376 excess perinatal deaths were associated with the first and second excess terms, respectively. The estimated relative increase in 1987 was 3.5% ± 0.9% (p = 0.0011), corresponding to 1,603 excess perinatal deaths (90% CI: 706–2,511). Fig 13 shows the trend in perinatal mortality rates and Fig 14 shows the residuals. The regression results (i.e., parameter estimates and standard errors of the estimates) are presented in Table 7, together with the results for Ukraine and Belarus.

**Table 7:** Regression results for Ukraine, Belarus, and Russia using Model 1.

Parameter	Variable	Ukraine		Belarus		Russia	
		Estimate	SE^1^	Estimate	SE^1^	Estimate	SE^1^
β_1_	intercept	−3.969	0.039	−4.461	0.062	−4.028	0.028
β_2_	t	−0.026	0.009	0.059	0.013	0.017	0.005
β_3_	t2	−1.3E-03	5.5E-04	−6.7E-03	6.9E-04	−2.4E-03	2.6E-04
β_4_	t3	3.6E-05	9.9E-06	1.1E-04	1.2E-05	2.6E-05	4.0E-06
β_5_	magnitude	1.496	0.155	4.320	0.477	0.482	0.170
β_6_	mu (μ)^2^	2.448	0.009	2.746	0.018	2.616	0.020
β_7_	sigma^3^	0.121	0.008	0.146	0.017	0.052	0.012
β_8_	magnitude	2.997	0.241	4.385	0.982	1.055	0.216
β_9_	mu (μ)	2.858	0.009	3.132	0.037	2.849	0.012
β_15_	d87	0.042	0.009	0.025	0.023	0.035	0.009

^1^SE: standard error of estimate;

^2^mu: ln(median),

^3^sigma: standard deviation of lognormal distribution

#### Regression with Model 2.

**Fig 15:**
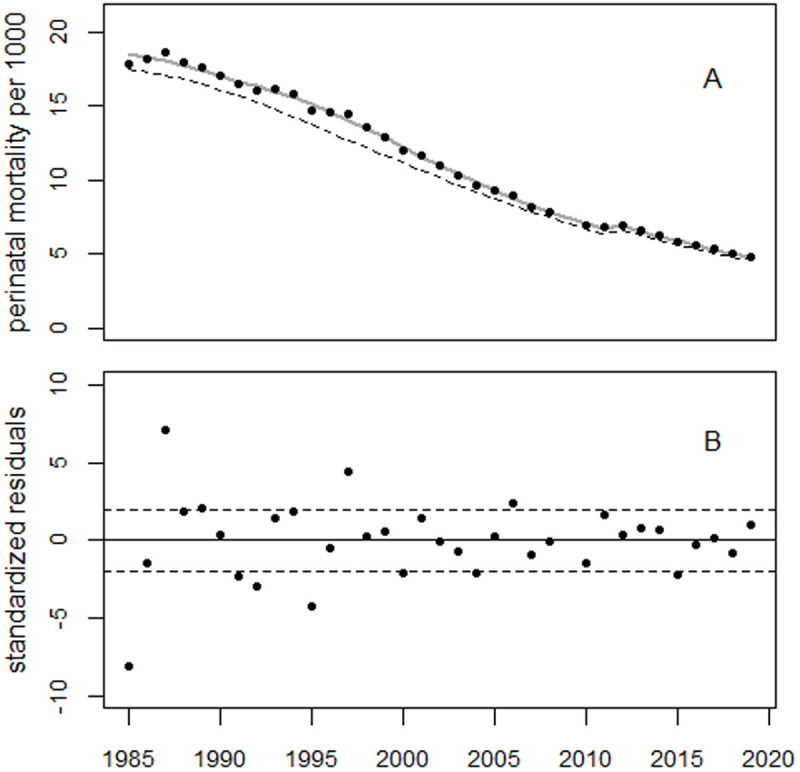
Perinatal mortality trend in the Russian Federation and the result of regression with only the GDP term. The dashed line shows the calculated trend without the impact of the GDP term Panel B: Residuals in units of standard deviation.

Regression of perinatal mortality rates with the inverse of GDP per capita (GDP term) reduced the deviance from 142.7 (df = 27) to 98.2 (df = 26), p = 0.002. Fig 15 shows the trend of perinatal mortality in Russia and the residuals. Note the highly significant increase in 1987. Adding the strontium term for age 15 at exposure—equivalent to age 14 when the maternal age distribution is shifted down by one year—reduced the deviance from 98.2 to 94.4 (p = 0.32). The estimate of the strontium term was negative.

### Moldova

#### Regression with Model 1.

In the HFA database, perinatal mortality data for birth weights of 1000 g or more since 1985 are available only for a few countries of the former Soviet Union, including Moldova and the Baltic States. These countries are far from the Chernobyl site, so strontium soil contamination should be negligible. Therefore, no effects of strontium on perinatal mortality are expected in these countries.

To analyze the data from Moldova, Regression Model 1 was supplemented with dummy variables for the years 1987 and 1988. The effect of the two bell-shaped excess terms was highly significant, reducing the deviance from 76.4 (df = 29) for the reduced model to 19.5 (df = 24), p = 1.8E-6.

A closer look at the data revealed a third peak around 2008. A regression model with a third bell-shaped term yielded a deviance of 11.8 (df = 22), indicating a significant improvement in fit compared to the model with two excess terms (p = 0.004).

**Fig 16:**
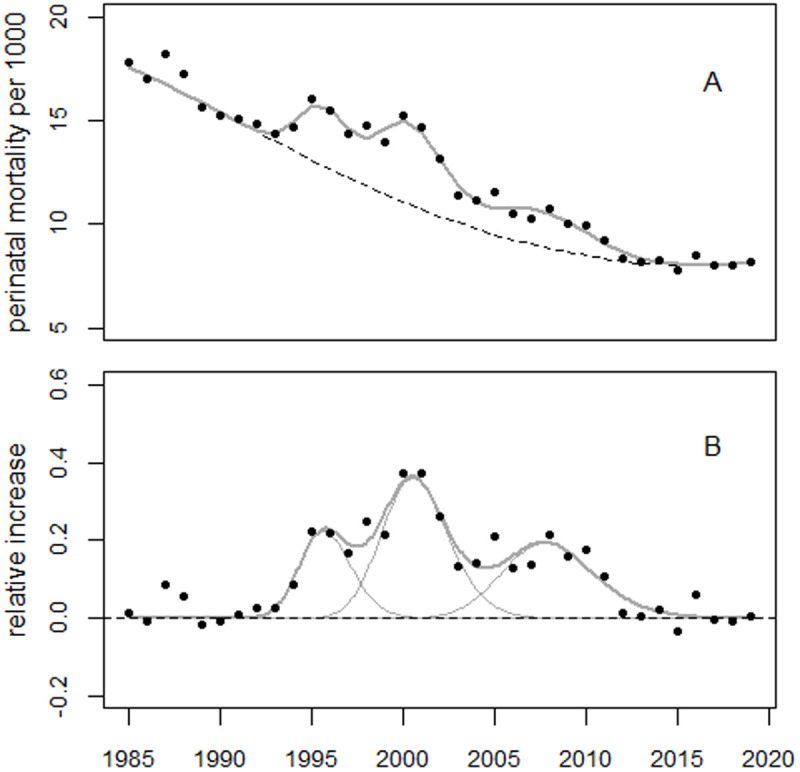
Panel A: Perinatal mortality trend in Moldova and regression line. The dashed line shows the predicted unperturbed trend. Panel B: Deviations from the trend in relative units.

**Fig 17:**
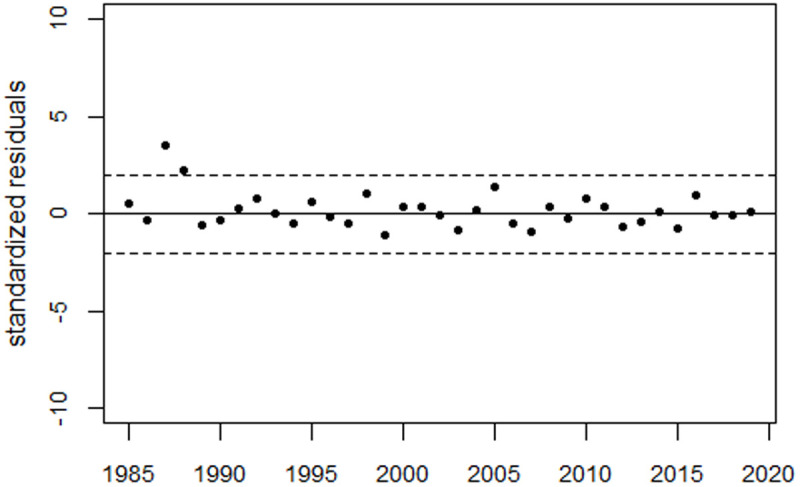
Deviations between observed and fitted perinatal mortality rates from Moldova in units of standard deviation (standardized residuals).

The maxima of the excess terms were found in 1995.6, 2000.5, and 2007.7. Peaks in 1987 and 1988 were statistically significant, with increases of 8.8% (p < 0.001) and 5.8% (p = 0.011), corresponding to 136 (90% CI: 79–194) and 85 (90% CI: 31–139) excess perinatal deaths, respectively. Figs 16 and 17 show the trend in perinatal mortality in Moldova and the residuals.

#### Regression with Model 2.

**Fig 18:**
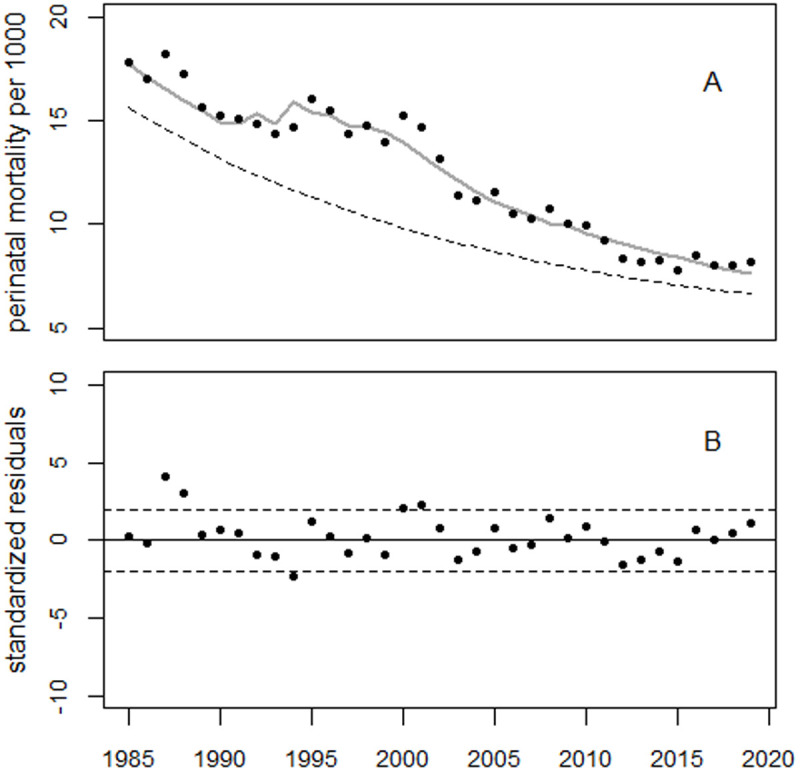
Panel A: Trend in perinatal mortality in Moldova and the result of regression with the GDP term. Panel B: Residuals in units of standard deviation.

Logistic regression of the Moldovan data with the GDP term reduces the deviance from 75.4 (df = 29) to 35.4 (df = 28), p < 0.0001. The model fits the data well (see the trend of residuals in Fig 18 panel B). Adding the strontium term for age 15 and at exposure does not significantly improve the fit (p = 0.074), and the strontium term estimate has a negative sign.

### Estonia

#### Regression with Model 1.

**Fig 19:**
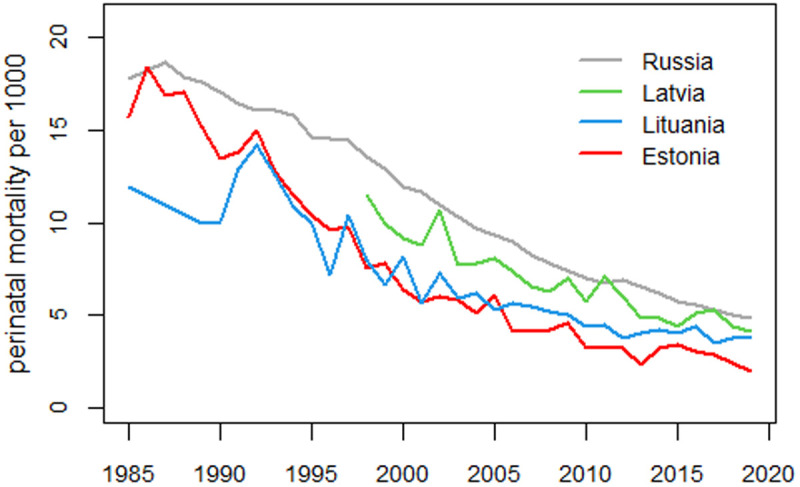
Perinatal mortality trends in the three Baltic countries and Russia.

Data on perinatal mortality in the three Baltic countries is also available in the HFA database. Fig 19 shows trends in perinatal mortality in Estonia, Latvia, Lithuania, and Russia. However, data from Latvia are only available from 1998 onwards, and the level of perinatal mortality in Lithuania from 1985 to 1990 is much lower than in Estonia. This raises doubts about the quality of the data, so only Estonia is considered in this study.

Soil deposition of strontium from the Chernobyl fallout should be negligible in the Baltic countries. Therefore, any increase in perinatal mortality before 1990 must be due to imported contaminated food. In her book Chernobyl: The Forbidden Truth, Ukrainian journalist Alla Yaroshinskaya published secret Politburo protocols from after the Chernobyl accident [14,14,15]. The following is an excerpt from [15]  of Protocol No. 32, dated August 22, 1986: “The Minister of Health of the USSR recommends distributing the contaminated meat as widely as possible in the country and using it to make sausages, preserved meat, and meat products in a proportion of one to ten with normal meat. In order to do this, it will be necessary to process it in factories in most areas of the Russian Federation (except Moscow), Moldavia, and the republics of Transcaucasus [sic], the Baltic States, Kazakhstan, and Central Asia”. Thus, the entire Soviet Union, except Moscow, was exposed to contaminated food products in 1987 and perhaps 1988.

**Fig 20:**
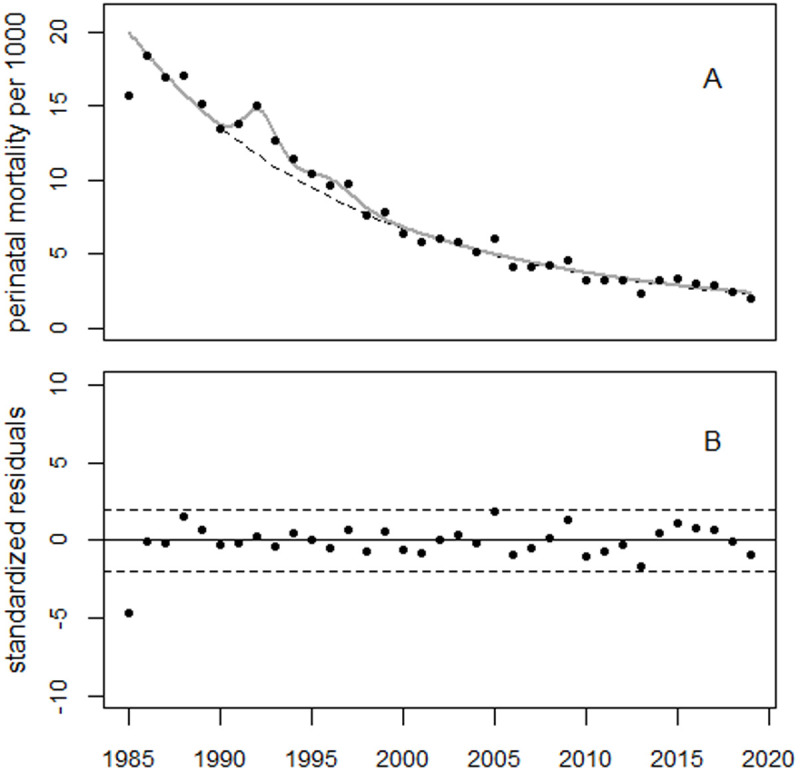
Trends in perinatal mortality in Estonia, as well as the results of a regression analysis using Model 1 (panel A) and standardized residuals (panel B).

To analyze the Estonian data, the cubic time trend was replaced with a linear-quadratic trend. Dummy variables were added for the years 1985 and 1988. Regression analyses with and without the two bell-shaped excess terms yielded deviances of 17.8 (df = 25) and 34.6 (df = 30), respectively. Thus, the effect of the excess terms is statistically significant (p = 0.004). The maxima of the bell-shaped terms were observed in 1992 and 1996, with increases of 26% and 14%, respectively. The 1985 data point is an outlier (p = 0.0001): it is 21% lower than the long-term trend would predict. The first peak after Chernobyl was observed in 1988 (+8%, p = 0.106). Fig 20 shows the trend in perinatal mortality and the residuals.

#### Regression with Model 2.

**Fig 21:**
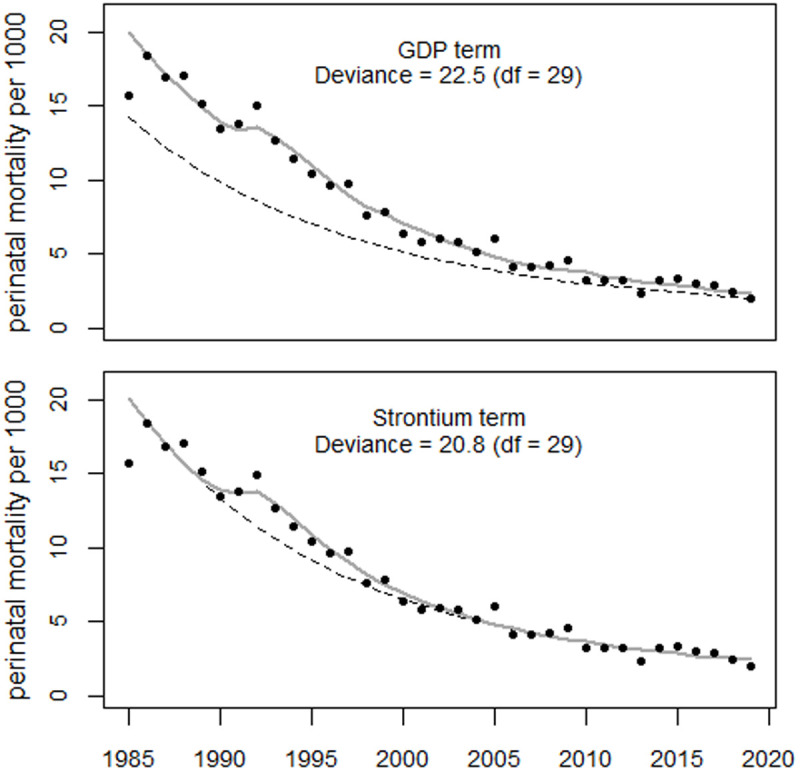
Trend in perinatal mortality in Estonia and the results of regressions using only the inverse GDP per capita (upper panel) and only the strontium term (lower panel) as a covariate.

Fig 21 shows the trend of perinatal mortality in Estonia and the regression results using Model 2. The model fits the data well. The deviance was 22.5 (df = 29) and 34.1 (df = 30) with and without the GDP term, respectively (p = 0.0006, F-test). Regression analysis using only the strontium term for ages 14, 15, and 16 at the time of exposure in 1987 produced deviance values of 24.7, 21.8, and 24.6, respectively. The corresponding p-values were 0.0026, 0.0004, and 0.0024, respectively. Thus, age 15 at exposure yielded the best fit, equivalent to an age distribution shifted down by one year and an age of 14 at exposure.

**Fig 22:**
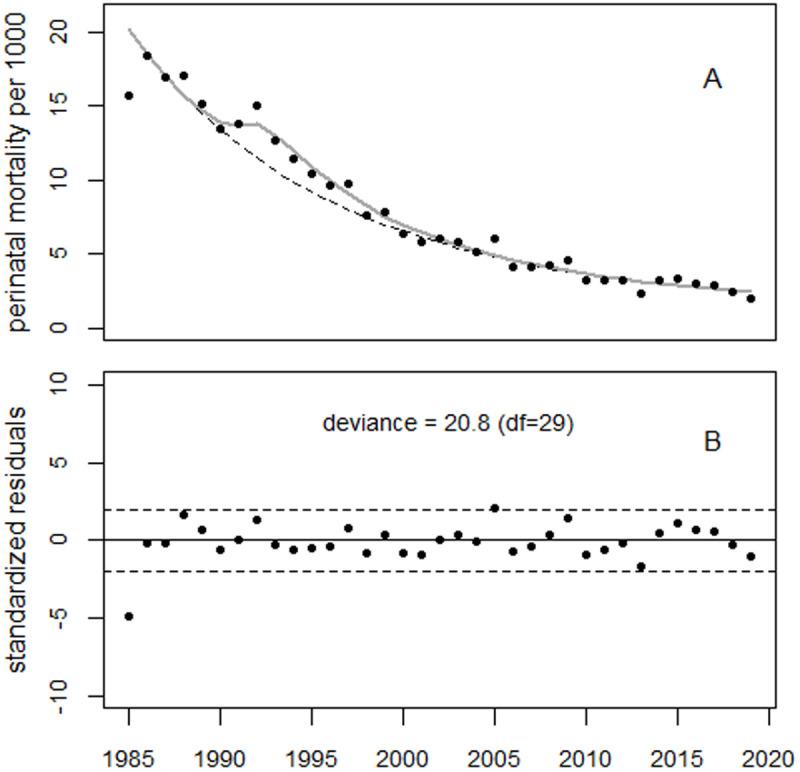
Trend in perinatal mortality in Estonia and the result of regression using only the Sr term to the power of two. Panel B shows the standardized residuals.

Regression with both the GDP and strontium terms yielded a deviance of 21.6 (df = 28) which was no notable improvement in fit over the model with only the strontium term (p = 0.59). Regression with the GDP term and the strontium term to the power of two reduced the deviance to 20.6. However, the GDP term had a negative sign. The best fit to the data was therefore obtained using only the strontium term to the power of two, yielding a deviance of 20.8 (df = 29); see Fig 22.

The strontium term was associated with 233 excess perinatal deaths, whereas the estimated increase of 8.4% in 1988 (90% CI: 0.5% to 15.8%) corresponds to only 33 excess deaths (90% CI: 2–68).

## Discussion

This study used the method described in [1] to analyze perinatal mortality trends after Chernobyl in Belarus and Russia. For comparison, data from Moldova and Estonia were also analyzed. The regression model consists of a long-term secular trend superimposed with two bell-shaped excess terms (Model 1). A second approach modeled the increase in perinatal mortality in the 1990s using the inverse of GDP per capita as a proxy for the impact of the socioeconomic crisis that followed the collapse of the Soviet Union in 1991 and the strontium concentration in pregnant women calculated with the maternal age distribution. Annual maternal age distribution data was unavailable for the countries studied, but the author had obtained age distributions from St. Petersburg.

Regressions with Model 1 fit the data well in all five countries. The regression results (i.e., the parameter estimates with standard errors) are shown in Table 7 for the three core countries: Ukraine, Belarus, and Russia.

The increase in the 1990s was greater in Belarus than in Ukraine, and much greater than in Russia. Compare the values for the variable magnitude. The variable mu (μ) is the natural logarithm of the median of the of the lognormal function. The variable sigma denotes the standard deviation, which relates to the half widths of the bell-shaped curves. As the effect size (magnitude) increases, the values of sigma increase. In Russia, the bell-shaped curves are narrower than in Belarus and Ukraine.

Table 8 shows the deviances for regressions with and without the two excess terms. Here dev1 and dev0 are the deviances for the full and the reduced models, respectively, while df1 and df0 are their respective degrees of freedom. The dispersion factor (OD) is the ratio of dev1 to df1, a measure of the goodness of fit of the full model. The p-values indicate the statistical significance of the improvement in the fit of the full model over the reduced model, i.e., the model without the bell-shaped excess terms.

**Table 8:** Significance of the bell-shaped excess terms from regressions with Model 1.

Country	dev0	df0	dev1	df1	OD1	F-value	p-value
Ukraine	176.2	20	10.7	15	0.71	46.49	<0.0001
Belarus	127.6	29	23.9	24	1.00	20.78	<0.0001
Russia	143.6	27	64.5	22	2.93	5.39	0.0022
Moldova	76.4	29	19.5	24	0.81	14.01	<0.0001
Estonia	34.6	30	17.8	25	0.71	4.72	0.0036

^1^OD (overdispersion): Ratio of dev1 to df1

Table 9 summarizes the effect of the inverse of GDP on the goodness of fit from regressions using Model 2 with only the GDP term. In all five countries, the effect is statistically significant.

**Table 9:** Regression using Model 2: Significance of the inverse of GDP as a covariate.

Country	dev0	df0	dev1	df1	OD	F-value	p-value
Ukraine	175.5	20	121.6	19	6.40	8.42	0.009
Belarus	114.0	28	29.2	27	1.08	78.41	<0.001
Russia	142.7	27	98.2	26	3.78	11.78	0.002
Moldova	75.4	29	35.4	28	1.27	31.59	<0.001
Estonia	34.1	30	22.5	29	0.78	14.97	0.001

Table 10 shows the improvement in fit when the strontium term is added as a second covariate to the GDP term. In all five countries, an age of 15 years at exposure in 1986 (1987 in Estonia) is used, which is equivalent to an age of 14 years at exposure when the age distribution is shifted down by one year. In Belarus, Russia, and Moldova, the strontium term estimates are negative. In Ukraine, the strontium term had a statistically significant effect. In Estonia, the strontium term alone explains the data trend; adding the GDP term does not significantly improve the fit (p = 0.59) and has a negative sign. A curvilinear dependence on strontium exposure with a strontium power of 2 improved the fit.

**Table 10:** Improvement in fit from the strontium term when added to the GDP term.

Country	dev1	df1	dev2	df2	OD	F-value	p-value
Ukraine	121.6	19	87.3	18	4.85	7.07	0.016
Belarus	29.2	27	26.8	26	1.03	2.33	0.139
Russia	98.2	26	87.8	25	3.51	2.96	0.098
Moldova	35.4	28	31.4	27	1.16	3.47	0.074
Estonia	22.5	29	21.6	28	0.77	1.17	0.289

Table 11 shows the increase in perinatal mortality in 1987, as well as the estimated number of excess perinatal deaths and their 90% confidence limits. The relative increases are similar in size in Ukraine, Belarus, and Russia (see column O/E), but are about twice as great in Moldova and Estonia. Table 11 shows two-tailed p-values; one-tailed p-values are half as large. For the number of excess perinatal deaths, 90% confidence intervals are reported.

**Table 11:** Relative increases in 1987, observed (O) and predicted (E) cases and 90% CI.

Country	O	E	O/E	O-E	CIL^1^	CIH^1^	p-value^2^
Ukraine	11823	11336	1.043	487	296	682	0.0004
Belarus	2206	2127	1.037	79.5	2	158	0.088
Russia	47081	45478	1.035	1603	706	2510	0.0011
Moldova	1689	1544	1.094	145	75	216	0.0011
Estonia^3^	431	398	1.084	33	2	68	0.089

^1^CIL, CIH: Lower and upper limits of 90% CI;

^2^two-tailed p-values;

^3^increase in 1988 in Estonia

A significant 6.5% increase in perinatal mortality was observed in Belarus in 1990–1991. During that same period, a highly significant 24% increase was observed in the Gomel Oblast. A highly significant peak was also found in Ukraine in 1991, in addition to the effect of GDP. These peaks occurred too early to be attributed to the socioeconomic crisis.

In Ukraine, the estimated number of excess perinatal deaths associated with the 1991 peak was 2,845. In 1987, the number of excess cases assumed to be due to cesium exposure was 487 (see Table 11). Thus, the excess perinatal deaths attributed to strontium exposure are approximately 5.8 times greater than those attributed to cesium exposure. Similarly, in Estonia, 233 excess cases were attributed to strontium exposure, while only 33 were attributed to cesium exposure—a ratio of 7.1.

The main merit of this study is that it was conducted. Nearly 40 years after the Chernobyl accident, no official study on perinatal mortality in the most contaminated countries of the former Soviet Union has been published in peer-reviewed English literature. A Google Scholar search found a master’s thesis on perinatal mortality in Kazakhstan, but only for the period from 1999 to 2008 [16]. The thesis contains information about the differences in how live birth and stillbirth were defined in the Soviet Union and by the WHO. These differences can be ignored as long as the definition remains consistent throughout the study period. In Kazakhstan, the WHO definitions of live births and stillbirths (also referred to as late fetal deaths) were adopted in 2008. This increased perinatal mortality from 13.2 per 1,000 in 2007 to 22.7 per 1,000 in 2008. In the present study, possible changes in the definition of perinatal mortality are accounted for by level shifts.

A 2023 paper by Volosovets et al. shows plots of infant mortality, neonatal mortality, fetal deaths per thousand births, and perinatal mortality in Ukraine from 1991 to 2021 (see Fig 1 in [17]). The authors cite the HFA database as their data source. However, the HFA database only provides data for Ukraine until 2010. Additionally, the plotted perinatal mortality data does not align with the HFA database data. Furthermore, different trends are shown for “perinatal mortality” and “perinatal mortality for birthweight of 1000 g or more,” whereas the figures in the HFA database are identical. Perinatal mortality increased from less than 10 per 1,000 in 2000–26 per 1,000 in 2001, a 160% rise. The authors attribute this increase to a change in the definition of stillbirths, shifting from 28 weeks of gestation to 22 weeks. In Germany, the level shift in perinatal mortality due to the change in the stillbirth definition in 1994 was approximately 35% (calculation by the present author).

## Conclusion

The marked deviation of perinatal mortality rates from the long-term trend in the 1990s is associated with GDP per capita. The peaks in 1987 and 1988 are likely due to cesium from the Chernobyl fallout. The sharp increase observed at the beginning of the 1990s in Ukraine and Estonia may be a delayed effect of strontium exposure in pregnant women. However, this hypothesis cannot be tested because strontium measurements in pregnant women after Chernobyl were unavailable.
